# End-of-life care and hospital-acquired bloodstream infection

**DOI:** 10.1017/ash.2023.290

**Published:** 2023-09-29

**Authors:** Melanie Zarnoski, Patrick Burke, Steven Gordon, Joanne Sitaras, Thomas Fraser

## Abstract

**Background:** All critically ill patients are at risk for hospital-acquired bloodstream infection (HABSI). At any time, however, there is heterogeneity among patients in the ICU; some patients have the added complexity of end-of-life discussions. We sought to better understand the patients in our medical intensive care unit (MICU) with HABSIs that do and do not meet the NHSN definition for a central-line–associated bloodstream infection (CLABSI) event by evaluating for the presence of a do-not-resuscitate (DNR) order. **Methods:** The study was conducted at our 66-bed MICU at the Cleveland Clinic Main Campus between January 2021 and September 2022. Surveillance for HABSI to include determination of CLABSI is performed prospectively according to the NSHN definition. The electronic health record was queried for each patient with a HABSI for the presence of a DNR order. DNR orders were categorized as follows: prevalent (DNR orders present at the time of admission to the MICU), incident (orders entered after admission to the MICU), or no DNR (for patients without an order at any time during their MICU stay). For incident orders, time from order to HABSI was recorded. Time to event was calculated as days between ICU admission to HABSI. **Results:** During the observation period there were 36,477 MICU patient days and 4,815 admissions. There were 112 HABSIs, of which 48 (43%) were CLABSIs. Overall, 65 patients were categorized as incident DNR, 7 were categorized as prevalent DNR, and 40 were categorized as no DNR. For patients with an incident DNR order, 50 HABSIs occurred on the date of or before the order and 15 occurred after the order. In patients in whom HABSI occurred after the incident DNR order, the median number of days between DNR order and HABSI was 11 days (range, 1–69). **Discussion:** In our MICU, >50% of HABSIs and 60% of CLABSIs occurred in patients with a DNR order incident to their MICU stay. Interventions to prevent hospital-acquired bloodstream infection and the analysis of the events are inextricably linked to issues of end-of-life care for critically ill patients. Further exploration of patient characteristics easily obtainable from the EHR, such as DNR orders, is necessary to inform best practices for prevention and risk adjustment of bloodstream infection rates.

**Figure:**
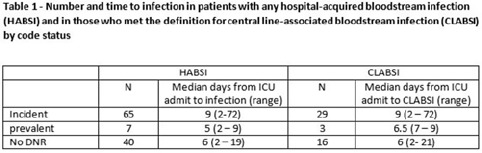

**Figure:**
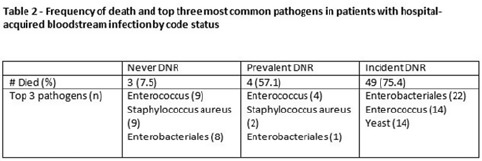

**Disclosures:** None

